# Cooking African Pumpkin Leaves (*Momordica balsamina* L.) by Stir-Frying Improved Bioactivity and Bioaccessibility of Metabolites—Metabolomic and Chemometric Approaches

**DOI:** 10.3390/foods10112890

**Published:** 2021-11-22

**Authors:** Petunia Mashiane, Vimbainashe E. Manhivi, Tinotenda Shoko, Retha M. Slabbert, Yasmina Sultanbawa, Dharini Sivakumar

**Affiliations:** 1 Department of Horticulture, Tshwane University of Technology, Pretoria 0001, South Africa; MashianeP@tut.ac.za (P.M.); SlabbertMM@tut.ac.za (R.M.S.); 2 Phytochemical Food Network Research Group, Department of Crop Sciences, Tshwane University of Technology, Pretoria 0001, South Africa; ManhiviVE@tut.ac.za (V.E.M.); ShokoT@tut.ac.za (T.S.); 3 Agricultural Research Council Industrial Transformation Training Centre for Uniquely Australian Foods, Queensland Alliance for Agriculture and Food Innovation, The University of Queensland, Brisbane, QLD 4072, Australia; y.sultanbawa@uq.edu.au

**Keywords:** β-carotene, phenolic compounds, traditional leafy vegetables, antioxidant activity, carbohydrate hydrolysing enzymes, bioaccessibility

## Abstract

The leaves of African pumpkins (*Momordica balsamina* L.) are a commonly consumed traditional vegetable. They are a good source of polyphenolic antioxidants and carotenoids, which are, however, affected by cooking or digestion. We investigated the effect of household cooking methods (stir-frying or boiling) on the changes in bioactive metabolites, antioxidant capacity, release and accessibility of β-carotene and also inhibition of inhibitory activity against α-amylase and α-glucosidase enzymes during in vitro digestion of African pumpkin leaves compared to the raw leaves. Compared to boiled or raw leaves, stir-frying improved the availability of bioactive metabolites at the gastrointestinal phase. Quercetin 3-galactoside and rhamnetin 3-O-glucoside (marker compounds) discriminated the stir-fried leaves from raw leaves and boiled leaves after digestion. Stir-frying improved the release and accessibility of β-carotene and enhanced the antioxidant activities compared to boiling. Dialysable fractions of stir-fried leaves exhibited the greatest inhibitory activity against α-amylase and α-glucosidase enzymes compared to the raw and boiled leaves, as well as acarbose. Stir-frying, therefore, is recommended for use in household cooking to benefit consumers by increasing the intake of phenolics and β-carotene.

## 1. Introduction

African pumpkins (*Momordica balsamina* L.) are tendril-bearing annual to perennial plants indigenous to tropical Africa and Asia [1]. African pumpkin is a wild leafy vegetable belonging to the Cucurbitaceae family. The plant is a miracle herb with enormous medicinal and nutritional properties [1] and reportedly contains polyphenolic antioxidants [2]. Lutein and β-carotene are the two most common carotenoids found in green leafy vegetables and exhibit antioxidant properties [3]. In African pumpkin, phenolic metabolites such as trans-4-p-coumaroylquinic acid, trans-4-feruloylquinic acid and cis-4-p-coumaroylquinic acid have been identified [4]. A common mechanism for phytochemicals to reduce oxidative stress is the ability to scavenge free radicals and ions [5]. This mechanism has been linked to the reduction in the risk of developing non-communicable diseases such as cardiovascular disease and diabetes [5]. Traditional diets of people of the Southern Africa region include African pumpkin leafy vegetables [6]. African pumpkin leaves are the cheapest source of micronutrients to support food security [6]. Therefore, the inclusion of this leafy vegetable in the daily diet needs promoting as the most practical and sustainable way to benefit consumer health.

Furthermore, this plant is drought-resistant, affordable, easy to grow and more accessible than exotic leafy vegetables such as spinach and cabbage [6]. Commonly, the consumption of African pumpkin leaves is in their cooked form through different cooking techniques (thermal processing methods), such as boiling, steaming, microwaving and stir-frying [7]. Rural people consume boiled leaves [7]. Cooking stops the browning enzyme action, reduces or eliminates the bitterness of vegetables and acid components and makes them palatable [8]. Studies have shown the effect of different cooking methods on phenolic compounds, carotenoids and antioxidant activity in leafy vegetables [7,9,10]. Cooking alters the chemical composition of vegetables, decreasing their bioaccessibility and number of bioactive compounds [11]. Moyo et al. [12] showed that there was no significant difference in raw or boiled African pumpkin leaves in terms of total phenolics. However, our previous study showed that stir-frying reduced the loss of flavanoids and improved the palatability of African pumpkin leaves, steaming reduced the loss of total phenolics and boiling reduced the total phenolics significantly [7]. However, the total phenolic compounds and other antioxidants present in food do not always reflect how much is available for the human body to absorb and metabolise [5].

In order to be absorbed into the circulatory system, phenolic compounds and carotenoids in vegetables need to be released from the plant matrix and solubilised into the gastrointestinal digesta through the in situ or ex situ effects (bioaccessibility) [13]. The concentration of phenolics and carotenoids bioaccessible in the intestinal tract may differ from that of the undigested leafy vegetable [5]. The information on the effects of cooking on different phenolic components, carotenoid components and the antioxidant activity of traditional leafy vegetables during intestinal digestion and bioaccessibility is limited. Furthermore, in vitro gastrointestinal digestion is technically easy to implement and requires no ethical approval. In this study, we investigated the effects of household cooking methods, stir-frying and boiling, on the changes in phenolic compounds, antioxidant activities, release, bioaccessibility of β-carotene and inhibition of carbohydrate hydrolysing enzymes during simulating gastrointestinal digestion compared to the raw leaves.

## 2. Materials and Methods

### 2.1. Chemicals, Reagents and Equipment

All chemical reagents, standards and solvents were purchased from Sigma Aldrich (Johannesburg, South Africa). All chemical reagents used were analytical grade.

All centrifugation was performed using a Hermle Z326k centrifuge (Hermle Labortechnik GmbH, Wehingen, Germany). All spectrophotometric determinations were performed using a microplate reader (BMG LABTECH GmbH, SpectroStar Nano, Ortenberg, Germany). Disease and decay-free African pumpkin leaves (20 kg) were selectively harvested twice according to maturity stage after 95 days of propagation from the vegetable garden belonging to the Zithobeni community (Bronkhorstspruit, South Africa). The leaves were cleaned by washing in running tap water and were transported to the laboratory at 10 °C within 2 h in cooler boxes and were stored at −80 °C for 48 h prior to cooking.

### 2.2. Household Cooking Techniques

African pumpkin leaves were cooked by following the methods described by Mashiane et al. [7].

Boiling: In a covered stainless-steel pot, 100 g of leaves (in 150 mL of water) were heated at 98 °C for 15 min to mimic the traditional method for boiling leaves. The leaves were drained once cooked and rapidly cooled on ice.

Stir-frying: In a pre-heated pan with olive oil (10 mL), 100 g of vegetables were stir-fried for 1–2 min. During the stir-frying, the oil temperature reached 130 °C, and the temperature of vegetables reached 100 °C. The samples cooled rapidly after being placed on ice.

A food thermometer probe (Mingle Development Co., Ltd., Tong Fu Yu Industrial Park, Shenzhen, China) was used to measure samples of various household cooking techniques [7]. Ten independent samples (weighing 100 g each) were collected from each cooking method, and 10 replicate samples of raw fresh leaves (control) were freeze-dried and stored at −80 °C for biochemical analyses. A batch of uncooked (raw) African pumpkin leaves and cooked leaves, as reported by Mashiane et al. [7], were stored at −80 °C prior to freeze drying. The samples were freeze-dried using a Benchtop Freeze dryer (VirTis Sp Scientific, Model #2kbtes-55 Gardiner, NY, USA) at –47 °C to –53 °C for 7 days. The freeze dryer was maintained at pressures below 200 millitorr. The fresh weight of the samples was recalculated based on the moisture content lost during freeze drying.

### 2.3. Simulated Gastrointestinal Digestion

The Infogest static in vitro simulation of gastrointestinal food digestion procedure according to Brodkorb et al. [14] and Seke et al. [15] was performed. An amount of 10 mL of simulated salivary fluid at pH 7 containing 75 U mL^−1^ α-amylase enzyme was added to 10 g of African pumpkin leaves, and the mixture was homogenised with a pestle and mortar for 10 s to mimic chewing then incubated in a shaking water bath at 170 rpm for 2 min at 37 °C. The simulated gastrointestinal fluids were as described before [15]. Simulated gastric fluid (20 mL) was added to the oral digesta, and the gastric phase was initiated, adjusting the pH to 2.5 using 6 M HCl, then adding pepsin solution (2000 U mL^−1^ in 0.1 M HCl, pH 2.2). The mixture was stirred at 170 rpm for 2 h at 37 °C; 10 mL samples were collected and cooled on ice for 10 min to stop reactions and then stored at 80 °C. In order to initiate the intestinal phase with dialysis, simulated intestinal fluid (20 mL) was added to the remaining gastric digesta, and the pH was adjusted to 7.5 using 2 M NaOH. A dialysis tube (10 cm, mw cut-off 10–12 kDa) was filled with 5 mL NaCl (0.9%) and 5 mL NaHCO_3_ (0.5 M) then placed inside the flask before adding 1.75 mL of a pancreatin solution (800 U mL^−1^), bovine bile extract and porcine bile extract (1:1 *w*/*w* up to 10 mM total bile salts) and 14 µL of 0.3 M CaCl_2_. The mixture remained under agitation at 37 °C for 2 h at 170 rpm. Digestion ended by cooling the samples in an ice bath and freezing all samples at −80 °C before analysis. A digestion blank without a sample was also kept. The determination of the bioaccessibility of β-carotene determined, according to Eriksen et al. [5], was by centrifuging instead of dialysing the digesta from the intestinal phase at 5000 rpm for 10 min at 4 °C. Separation and centrifugation were repeated, and the supernatant was kept as the bioaccessible fraction and then freeze-dried. For this study, the dialyzed compounds represent the material that enters the serum, while the solution remaining outside the membrane represents the material passed into the lumen, as described by Bermúdez-Soto et al. [16].

### 2.4. Extraction of Phenolic Metabolites

Samples of freeze-dried African pumpkin leaves (50 mg) were dissolved in ethanol/water (70:30, *v*/*v*), ultrasonicated for 30 min and centrifuged at 1000× *g* for 20 min at 4 °C. The extraction was repeated, and the supernatants were pooled together before filtering using polytetrafluorethylene filters, as described by Managa et al. [10].

The samples were run by using ultra-performance liquid chromatography (UPLC) with a Water Acquity photodiode array detector (PDA) coupled with a Synapt G2 quadrupole time-of-flight (QTOF) mass spectrometer (MS)(Waters, Milford, MA, USA), as previously described by Managa et al. (2020), without any modifications. UPLC-Q-TOF/MS analyses were subject to a series of procedures, including baseline correction, denoising, smoothing, time-window splitting, deconvolution and peak alignment. The raw abundance (peak areas of the metabolites) and retention time (RT) of dialysable fractions of raw, stir-fried or boiled African pumpkin leaves, as well as the *m*/*z* ratio, were generated and uploaded to MetaboAnalyst. Concentrations of phenolic compounds were reported as mg 100 g^−1^ sample.

### 2.5. β-Carotenoid Content

β-Carotenoid content was determined according to Panfili et al. [17], with a few modifications. Briefly, a portion (0.25 g) of each sample (freeze-dried leaves and digestive fractions) was extracted using 5 mL of acetone:hexane (1:1) with 0.1% butylated hydroxytoluene (BHT). The mixture was centrifuged at 3450× *g* for 15 min at 25 °C, and the supernatant was evaporated to dryness under a nitrogen stream. The extract was re-dissolved in 1 mL of isopropyl alcohol (10%) in *n*-hexane filtered using a PTFE filter before analysis. A 10 µL sample was injected, and separation of carotenoids was achieved using an isocratic elution of 10% isopropyl alcohol in *n*-hexane (*v*/*v*) for 30 min on a Shim Pack GIST column with dimensions of 250 mm × 4.6 mm i.d., 5 m particle size using a Shimadzu Prominence-i-LC-2030C 3D equipped with an Autosampler (SIL-20A) HPLC system (Shimadzu, Kyoto, Japan). Identification of β-carotene was based on a comparison of retention times with that of pure standard. The reference calibrant β-carotene (LOQ 6, 32, LOD 19, 16) was used to quantify β-carotene based on area. Carotenoid bioaccessibility was calculated as the ratio between carotenoid concentration in the micellar aqueous phase (supernatant) of the intestinal fraction and its initial concentration in the undigested sample.

### 2.6. Antioxidant Capacity

A radical scavenging assay was conducted using the 2,2-diphenyl-2-picrylhydrazyl (DPPH) method previously described by Seke et al. [15]. The reaction mixture contained 250 µL of DPPH (90 mM) solution and 20 µL of the sample (various concentrations); the absorbance read 517 nm. The results were expressed as IC_50_. The ABTS radical cation (ABTS^+.^) scavenging capacity was determined using a 40 µL sample (various concentrations) and 200 µL of ABTS^+^, the absorbance was measured at 734 nm, and the results were expressed as IC_50_ (mg mL^−1^).

The ferric reduction antioxidant capacity (FRAP) assay was conducted according to Seke et al. [15]. The reaction mixture included the 220 µL of FRAP reagent solution and 15 µL of the homogenised leaf extract; the absorbance was at 593 nm, and antioxidant activity was expressed as Trolox equivalent antioxidant activity (TEAC)100 g^−1^.

### 2.7. In Vitro α-Glucosidase Inhibitory Activity

The α-glucosidase inhibitory activity of extracts of cooked African pumpkin leaves was determined using the method described by Seke et al. [15]). The sample (50 µL different concentrations) and 100 µL of 0.1 M phosphate buffer (pH 6.9) containing α-glucosidase solution (1 U mL^−1^) were incubated at 25 °C for 10 min. Thereafter, 50 µL of 5 mM p-nitrophenyl-α-d-glucopyranoside in a 0.1 M phosphate buffer (pH 6.9) was incubated at 25 °C for 5 min. The absorbance measured 405 nm, and the inhibiting activity of α-glucosidase was as IC_50_.

### 2.8. α-Amylase Inhibitory Activity

As described previously by Moloto et al. [18], an assay was performed using porcine pancreatic α-amylase, incubating 500 μL of the sample (leaf extract) and 500 μL of 20 mM phosphate buffer (pH 6.9) with 6 mM NaCl containing α-amylase solution (0.5 mg mL^−1^) at 25 °C for 10 min. After incubation, 1% starch solution (500 µL) in 20 mM phosphate buffer (pH 6.9 with 6 mM NaCl) was incubated for 10 min at 25 °C. A KAT amylase reagent was added to 100 µL of reaction solution for 5 min. After cooling to 25 °C, the absorbance was at 540 nm. Acarbose was for comparison purposes. The IC_50_ value (g mL^−1^) for α-amylase inhibition was determined.

### 2.9. Statistical Analysis

This study employed a completely randomised design, with 10 replications per cooking technique and two repeated experiments. A one-way ANOVA analysed the differences between household cooking techniques using Genstat (VSN International, Hemel Hempstead, UK) for Windows 13th Edition (2010 version). The least significant difference test (LSD) was used to compare the means of the cooking treatments, at *p* < 0.05. Following the UPLC-Q-TOF/MS analysis, data files were subsequently imported into MetaAnalyst 5.0 for partial least squares discriminant analysis (PLS-DA), variables importance in projection (VIP) scores and heat maps. Furthermore, it is a statistical procedure to analyse the correlation of two quantitative variables. In their simplest form, correlation coefficients illustrate the relationship between two variables, while low correlation coefficients reveal the absence of a relationship [19]. Data were transformed according to Cahvallo et al. [19], and a correlation threshold of 0.5 was used for the correlation to be considered significant [19].

## 3. Results and Discussion

Supplementary Table S1 lists the 14 major bioactive metabolites detected in raw undigested and cooked (boiling and stir-frying) and digested African leaves by UPLC-QTOF/MS. Table 1 illustrates the influence of stir-frying and boiling household cooking methods during in vitro gastrointestinal digestion and the changes in bioactive metabolites present in African pumpkin leaves compared to whole raw leaves. In the raw, undigested leaves, the most abundant phenolic metabolite was cis-4-feruloylquinic acid (273.67 mg 100 g^−1^). African pumpkin leaves contain six different quinic acid derivatives, i.e., methylquinic acid, 4-caffeoylquinic acid (cryptochlorogenic acid), cis 4-coumaroylquinic acid, trans-4-coumaroylquinic acid, cis-4-feruloylquinic acid and trans-4-feruloylquinic acid, as well as pseudolaroside A, melilotoside, quercetin-3-rutinoside (rutin), nicotiflorin, keioside, rhamnetin-3-O-glucoside and phenethyl rutinoside.

Overall, cooking improved the bioaccessibility of most of the metabolites, except for methylquinic acid, cis-4-feruloylquinic acid and phenethyl rutinoside in African pumpkin leaves. Stir-frying improved the bioaccessibility of most bioactive metabolites in African pumpkin leaves compared to boiling and whole raw leaves. In contrast, the concentration of methylquinic acid and phenethyl rutinoside was significantly reduced during thermal cooking (stir-frying and boiling). Stir-frying can lead to an increase in 4-caffeoylquinic acid due to the isomerisation and transformation of 5-caffeoylquinic acid (chlorogenic acid) [20]. Managa et al. [10] also found 4-caffeoylquinic acid during all cooking methods, with stir-frying having the highest concentration. Due to isomerisation and transmutation of 5-caffeoylquinic acid, high temperatures also caused an increase in 4-O-caffeoylquinic acid levels in artichoke [21]. In contrast to Wang and Ho [22], who reported it was possible to produce caffeic acid by hydrolysis of caffeoylquinic acid, our results suggest that boiling removed caffeic acid, and there was no detection in stir-fried leaves. Similarly, thermal cooking reduced cis-4-feruloylquinic acid, with a similar observation during the roasting of coffee beans [23]. Overall, stir-frying improved the bioaccessibility of most of the bioactive metabolites in African pumpkin leaves in this study. Since stir-frying destroys cell walls and other subcellular components, most phenolic compounds can be released and retained on the leaf surface. In addition, the polarity of the medium may have greatly reduced the number of changes in phenolic compounds [24].

In general, different bioactive metabolites in gastrointestinal digesta of raw, stir-fried and boiled leaves increased significantly compared to their respective undigested counterparts. It is possible that phenolic compounds can be released from the vegetal matrix under the action of pepsin and the pH of the environment (acidic or alkaline) during digestion if the phenolics form complexes with proteins [25]. According to Tagliazucchi et al. [26], the release of phenolic compounds during intestinal digestion was probably due to exposure to acidic and alkaline conditions during simulated intestinal digestion hydrolyses bonds between these compounds.

These results confirm that both acidic and alkaline hydrolysis in the digestion phases seem to have a significant impact on phenolic compound stability. The concentration of methylquinic acid, phenethyl rutinoside and trans-4-feruloylquinic acid decreased significantly in the dialysable digesta of stir-fried and boiled African pumpkin leaves compared to the dialysable digesta of raw leaves. However, the concentrations of the majority of the identified bioactive metabolites (4 caffeoylquinic acid, cis 4-coumaroylquinic acid, trans-4-coumaroylquinic acid, quercetin-3-rutinoside, trans-4-feruloylquinic acid, quercetin 3-galactoside and isorhamnetin 3-O-robinoside, rhamnetin-3-O-glucoside) increased in the dialysable digesta of stir-fried African pumpkin leaves in comparison to the raw or boiled leaves. The chromatogram in Supplementary Figure S1 illustrates the increase in different bioactive metabolites in the gastric, intestinal and dialysable digesta of stir-fried African pumpkin leaves. Additionally, since olive oil is a nonpolar medium, it would have prevented the loss of phenolic compounds due to lack of diffusion and migration [27]. Furthermore, boiling led to the destruction of cell walls, which could explain the observed reduction in the concentration of bioactive metabolites caused by compounds leaching out into the water. Kaempferol-O-rutinoside levels remained at a higher concentration in the dialysable digesta of raw in comparison to stir-fried and boiled leaves. In the dialysable digesta of raw and boiled leaf, the concentration of the phenolic metabolite β-D-glucosyl-2-coumarate was higher than that in the digesta of stir-fried leaf. However, according to Rohn et al. [28], heat more readily degrades the sugar moiety attached to the C-ring of β-D-glucosyl-2-coumarate [28].

From this study, it is evident that stir-frying improved most bioactive metabolites compared to boiling. However, quercetin-3-rutinoside was very stable during digestion of raw and stir-fried leaves under pancreatic conditions, but it was also noted in boiled leaves. Bermúdez-Soto et al. [16] reported a similar stability of quercetin-3-rutinoside in chokeberry juice during intestinal digestion. This study also confirmed an increase in 4-caffeoylquinic acid (cryptochlorogenic acid) during intestinal digestion of raw, stir-fried and boiled African pumpkin leaves. Therefore, the obtained results did not agree with previous reports on the isomerisation of caffeoylquinic acid during pancreatic incubation [29].

For this study, the dialysed compounds represent the material that enters the serum, while the solution remaining outside the membrane represents the material passed into the lumen, as described by Bermúdez-Soto et al. [16]. Dialysis membranes, however, could have resulted in substantial losses of some phenolic compounds. The reasons for these losses were largely attributable to the difficulty of washing membranes and recovering compounds, as described by Bermúdez-Soto et al. [16]. The dialysis process is also complicated by factors such as the volume and composition of the buffer used, sugar concentration in the sample or the number of molecules that bind to the membrane during the process [16]. In the dialysable digesta of raw, stir-fried or boiled leaves, all these factors could have contributed to the bioaccessible amount of different phenolic compounds. Bioactive metabolites in intestinal digesta of raw, stir-fried and boiled African pumpkin leaves were higher than their respective dialysable digesta. Since most compounds were unable to cross the simulated intestinal barrier (the dialysable membrane) and would therefore not be absorbed, these compounds will enter the colon and be metabolised by colonic bacteria, promoting significant health benefits on the host [30].

Compared to undigested raw or boiled African pumpkin leaves, stir-fried leaves exhibited the highest (*p* < 0.05) FRAP, DPPH and ABTS activities. This may be due to the higher content of phenolics and carotenoids. FRAP, DPPH and ABTS antioxidant activities were higher in the dialysable digesta of stir-fried leaves compared to raw and boiled leaves (Table 1). This could be due to the presence of a higher amount of bioactive metabolites in the dialysable fraction observed in this study. This could also be due to certain compounds concentrated in this fraction that may have higher antioxidant activities. The antioxidant activity of pumpkin leaves was reported to be correlated to the concentration of phenolic compounds [31].

### 3.1. Multivariate Analysis

**Figure 1:**
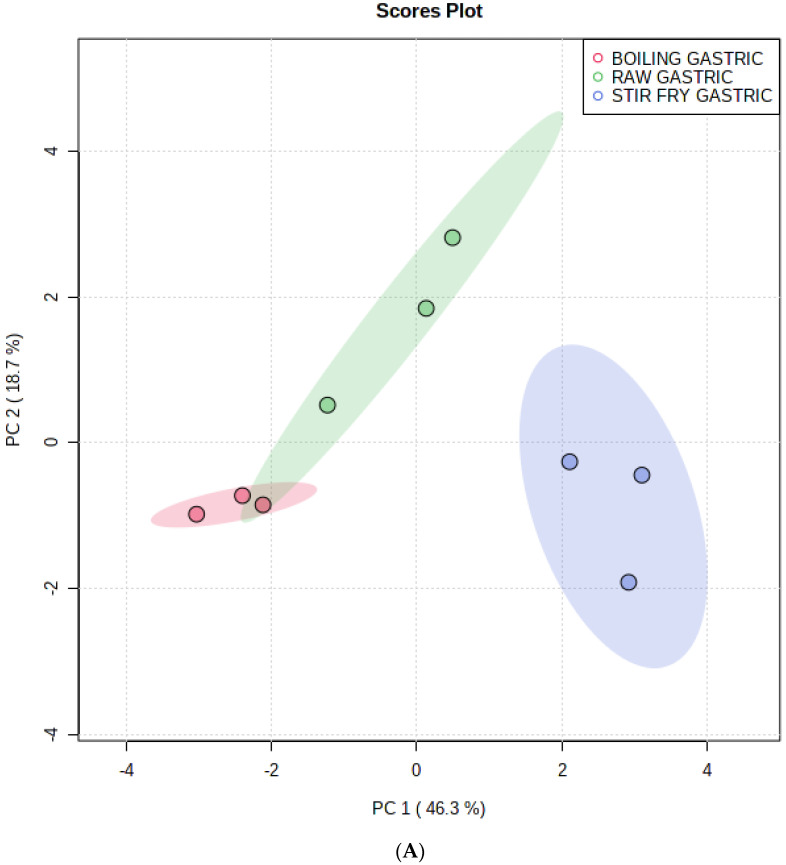

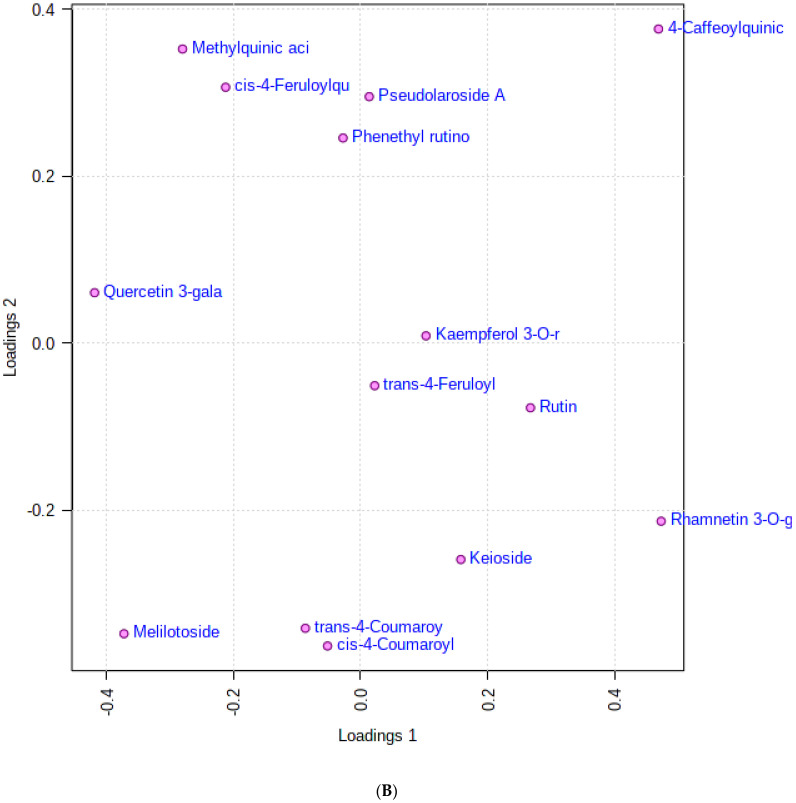

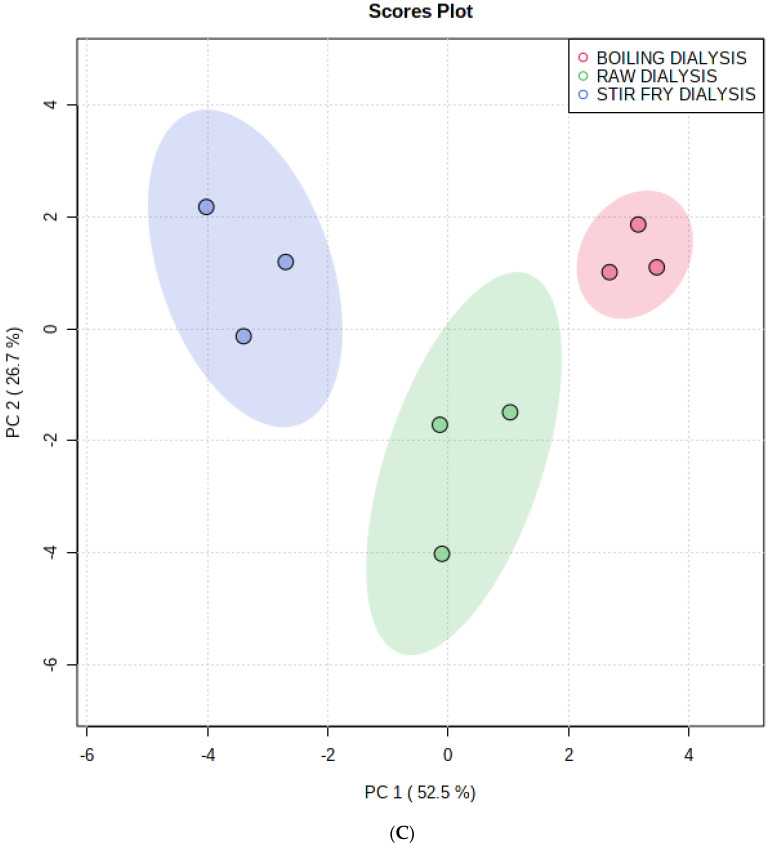

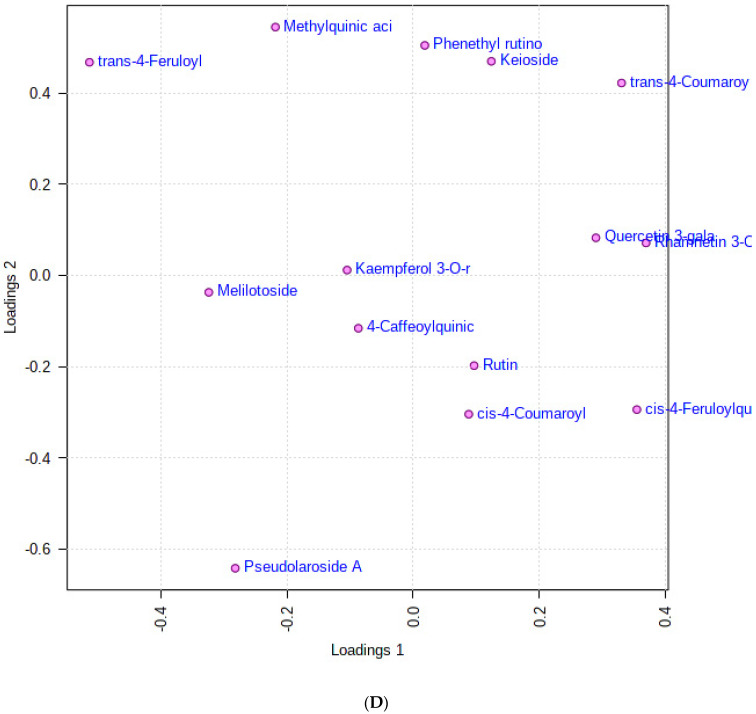

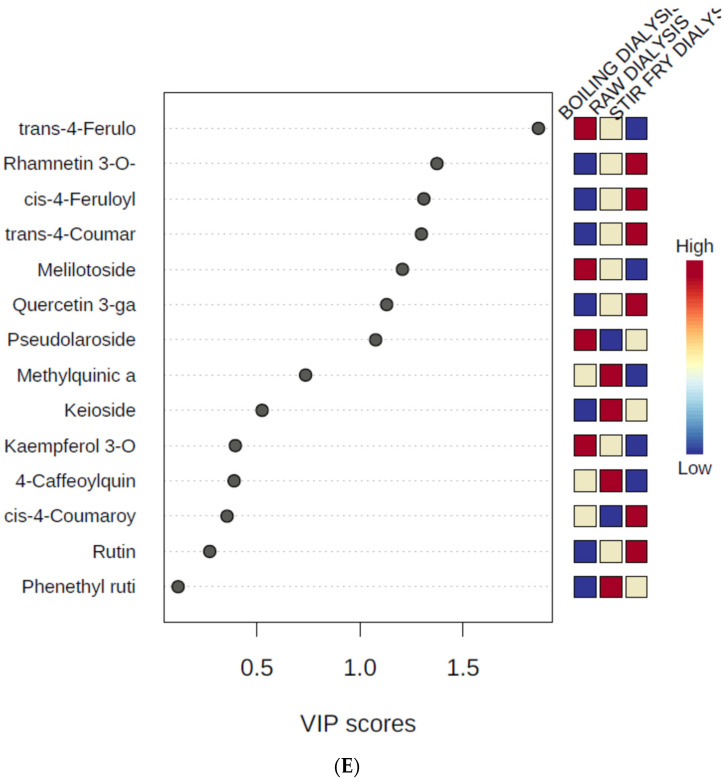

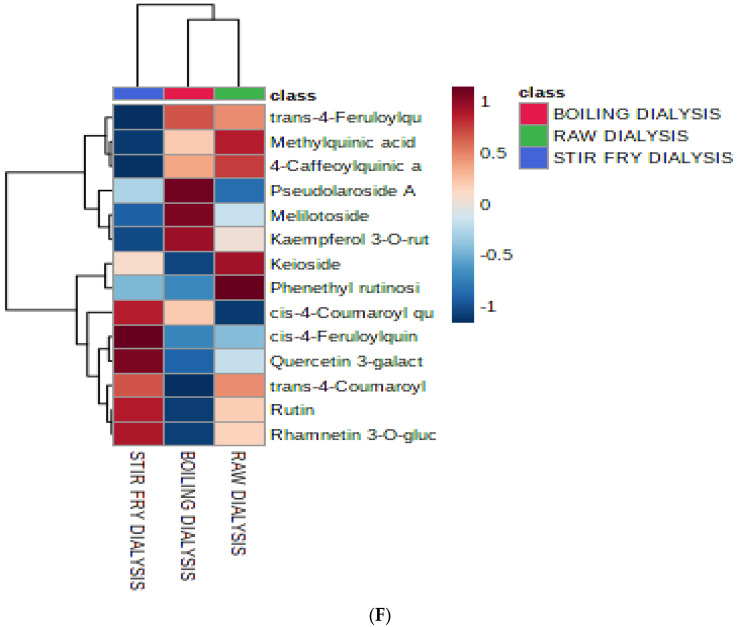

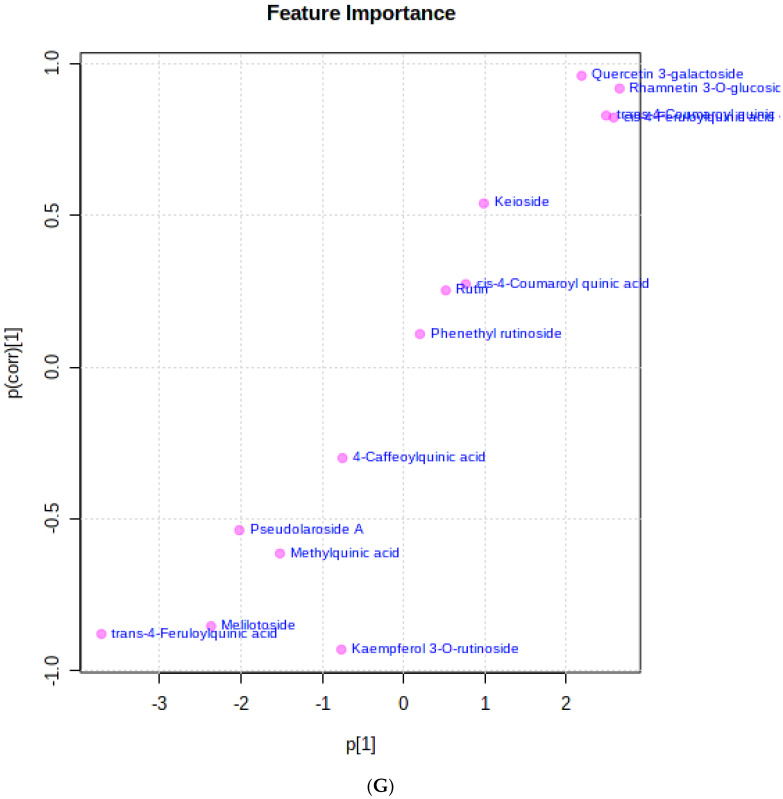
(**A**). Statistical analyses of bioactive metabolites, by using the Metaboanalyst 4.0 software, illustrating the unsupervised PCA score plot of bioactive metabolites of dialysable digesta of stir-fried, boiled or raw African pumpkin leaves generated by UPLC-QTOF/MS analysis and the separation of three clusters. (**B**) Illustration of the PC 1 and PC2 loadings of bioactive metabolites generated by the UPLC-QTOF/MS analysis from the dialysable digesta of stir-fried, boiled and raw leaves responsible for the separation of three different clusters. (**C**) Supervised PLS-DA score plot showing separation of three clusters for dialysable digesta of stir-fried, boiled and raw leaves based on bioactive metabolites generated by the UPLC-QTOF/MS analysis. (**D**) Illustration of the PC 1 and PC2 loadings of PL-SA analysis. Bioactive metabolites generated by the UPLC-QTOF/MS analysis from the dialysable digesta of stir-fried, boiled and raw leaves responsible for the separation of three different clusters. (**E**) VIP scores of bioactive metabolites in PLS-DA. The most abundant compounds in the dialysable digesta of stir-fried, boiled and raw African pumpkin leaves are represented by their m/z values, retention times (min). The scoring of the variables, ranging from low to high numbers, is according to their importance. On the right, the coloured boxes indicate the relative concentration of the corresponding metabolites. A red colour indicates high level, and a blue colour indicates a low level. (**F**) Heat map. The coloured areas on the map correspond to concentrations of different bioactive metabolites present in the dialysable digesta of stir-fried, boiled or raw African pumpkin leaves. Bioactive metabolites are in rows, and different household cooking techniques are in columns. The red colour indicates high levels and the blue colour low levels. (**G**) showing the OPLS-DA loadings S-plot and the compounds responsible for the separation of dialysable digesta of stir-fried leaves from the boiled and raw leaves.

A general picture of the relationship among the data matrix was obtained with MetaboAnalyst 4.0’s unsupervised principal component analysis (PCA). A supervised partial least-squares discrimination analysis (PLS-DA) evaluated differences in metabolite levels in dialysable fractions with raw, stir-fried or boiled African pumpkin leaves. It is crucial to take into account variables that are important to projections (VIP) when interpreting PLS-DA data. Those metabolites with VIP values > 1 were thought to play an important role in distinguishing the intestinal or dialysable fractions with variation in cooking methods (stir-fried, boiled leaves or raw). In this work, PLS-DA classified the stir-fried, boiled and raw African pumpkin leaves digested using the simulated dialysable phase based on the available bioactive metabolites in these samples. There was a comparison obtained of the metabolites in the dialysable fraction of stir-fried, boiled and raw leaves using unsupervised PCA (Figure 1A). According to the first and second principal components, there was an explanation for the PC 1 (46.3%) and PC 2 (18.7%) variance. Based on the bioactive metabolites in the dialysable phase, we observed a separation of three clusters for raw, stir-frying and boiling cooking methods in the unsupervised PCA plot. The PLS-DA model shows a good level of goodness-of-fit (R^2^ = 0.97) and has a high predictability level (Q^2^ = 0.91), which allowed us to forecast metabolite changes from the dataset. Figure 1B shows the loadings of different phenolic compounds on PC1 and PC2. The 4-caffeoylquinic acid (cryptochlorogenic acid) and rhamnetin-3-O-glucoside accumulated positively on PC1 and distinguished the stir-fried leaves from the boiled and raw leaves. Figure 1C presents the different clusters of dialysable digesta of raw, stir-fried and boiled African pumpkin leaves. Figure 1D shows that the loading of different metabolites on PLS-DA and trans-4-coumaroylquinic acid, quercetin 3-galactoside and rhamnetin-3-O-glucoside was responsible for the separation of stir-fried leaves from the boiled and raw leaves.

The basis for the ranking of metabolites was on their VIP scores, with only top-ranking metabolites with the highest VIP scores considered [31]. Among the top eight metabolites with VIP scores greater than 1 were trans-4-feruloylquinic acid, rhamnetin-3-O-glucoside, cis-4-feruloylquinic acid, trans-4-coumaroylquinic acid, β-D-glucosyl-2-coumarate (Melilotoside), quercetin 3-galactoside and pseudolaroside A (Figure 1E). In addition to this analysis, there was a calculation of a heat map from metabolite concentrations across all samples (Figure 1F). The heat map showed raw and cooked leaves, along with their corresponding bioactive phenolic metabolites, with dark red boxes indicating higher levels and dark blue boxes indicating lower levels. Based on the heat map, rhamnetin-3-O-glucoside, rutin, trans-4-coumaroylquinic acid, quercetin 3-galactoside, Cis-4-feruloylquinic acid and cis 4-coumaroylquinic acid were at higher concentrations in the dialysable fraction of stir-fried African pumpkin leaves. By using OPLS-DA loadings S-plot (Figure 1G), quercetin 3-galactoside and rhamnetin 3-O-glucoside are shown as the marker compounds that separated the stir-fried leaves from raw and boiled leaves at the dialysable phase. Trans-4-feruloylquinic acid separated the raw leaves from the cooked African pumpkin leaves.

### 3.2. Inhibition of Carbohydrate Hydrolysing Enzymes

The hydrolysis of carbohydrates to dextrin by pancreatic α-amylase activity, which in turn is hydrolysed further to glucose by intestinal α-glucosidase enzyme, causes postprandial hyperglycaemia. An important strategy in the management of type two diabetes is to inhibit these enzymes [14]. In this study, raw African pumpkin leaves showed the highest inhibition of α-amylase and α-glucose activities compared to the commercial anti-hyperglycaemic agent acarbose. All gastric, intestinal and dialysable fractions of raw, stir-fried or boiled leaves showed the highest inhibitory activity of both the carbohydrate hydrolysing enzymes. The dialysable fraction of stir-fried leaves, however, showed the highest inhibitory activity of amylase and glucosidase enzymes compared to all fractions of raw and boiled leaves and acarbose (Table 2). Due to their inhibitory effects, these enzymes delay carbohydrate digestion, which in turn leads to a reduction in the rate of glucose absorption after a meal, which ultimately reduces the postprandial rise in blood glucose levels [32]. The inhibitory effects of polyphenols on the enzymes α-glucosidase and α-amylase are associated with their structural features, which includes the position and number of hydroxyls and the number of double bonds on aromatic rings A and B as well as the heterocyclic ring C. Studies observed that flavonoids with double bonds between C-2 and C-3 and hydroxyls on the C6 and C7 positions of ring A and C-4 position of ring B in flavonoids show good α-glucosidase and α-amylase inhibition activity [33].

The findings of this study provide evidence-based information that could be used to promote the consumption of stir-fried African pumpkin leaves to alleviate postprandial hyperglycaemia. Additionally, Van Elslande [34] found that *Momordica charantia* inhibited both α-glucosidase and α-amylase enzyme activities, thereby hindering glucose digestion and absorption from the proximal intestine. Furthermore, cooking affected the inhibitory activity of carbohydrate hydrolysing enzymes differently in different vegetables. In vitro digestion improved the α-glucosidase activity of cooked mushrooms [35].

### 3.3. The Relationship between the Bioactive Metabolites Identified and the Tested Biological Activities

It is a statistical procedure to analyse the correlation of two quantitative variables. In their simplest form, correlation coefficients illustrate the relationship between two variables, while low correlation coefficients reveal the absence of a relationship [31]. Data were transformed according to Cahvallo et al. [31], and a correlation threshold of 0.5 was used for the correlation to be considered significant [36].

**Figure 2:**
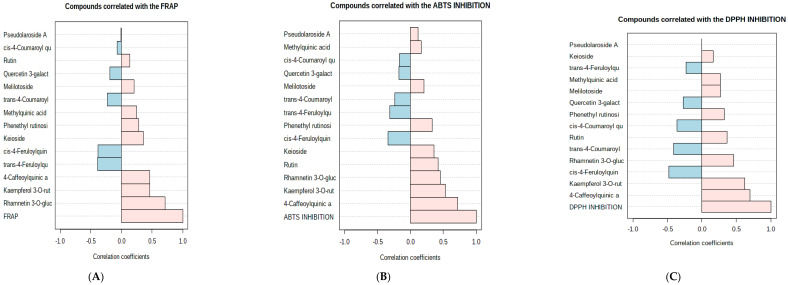

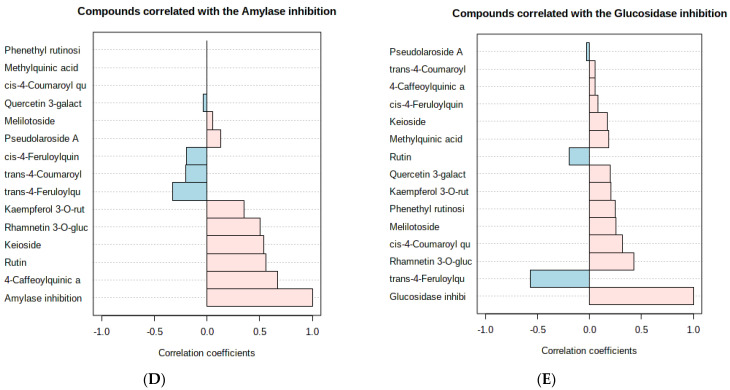
(**A**–**E**) Shows the correlations of metabolites involved in antioxidant activity, FRAP, ABTS, DPPH and inhibition of α-glucosidase and α-amylase.

Figure 2A–E shows the correlations of metabolites involved in antioxidant activity, FRAP, ABTS, DPPH and inhibition of glucosidase and amylase. Rhamnetin 3-O-glucoside showed a strong positive correlation with FRAP activity (r = 0.71444). Furthermore, the DPPH scavenging activity correlated positively with 4-caffeoylquinic acid (r = 0.70) and kaempferol 3-O-rutinoside (r = 0.62). Additionally, 4-caffeoylquinic acid (r = 0.71643) and kaempferol 3-O-rutinoside (r = 0.53) showed positive correlation with ABTS activity. Moreover, antioxidant metabolites’ activity is dependent not only on their concentration but also on their reactivity with reactive oxygen species. Since oxidising radical species readily attract hydroxyl groups, the phenolic groups and extensively conjugated electron system of metabolic derivatives with strong antioxidant activity facilitate electron transfer between the radical and hydroxyl groups [37]. It is likely that the active hydrogen donor capability of hydroxyl substitution was responsible for the observed scavenging activity [38].

With α-amylase 4-Caffeoylquinic acid (r = 0.67), rutin (r = 0.56), isorhamnetin 3-O-robinoside (Keioside) (r = 0.55) and rhamnetin 3-O-glucoside (r = 0.50) showed a positive correlation, while none of the compounds correlated positively with α-glucosidase activity. There is a possibility that other compounds, such as alkaloids, terpenes or peptides, can show an inhibitory effect on α-glucosidase [39].

### 3.4. Impact of Stir-Frying or Boiling on the Release and In Vitro Bioaccessibility of β-Carotene Compared to the Raw Whole Leaves

**Figure 3:**
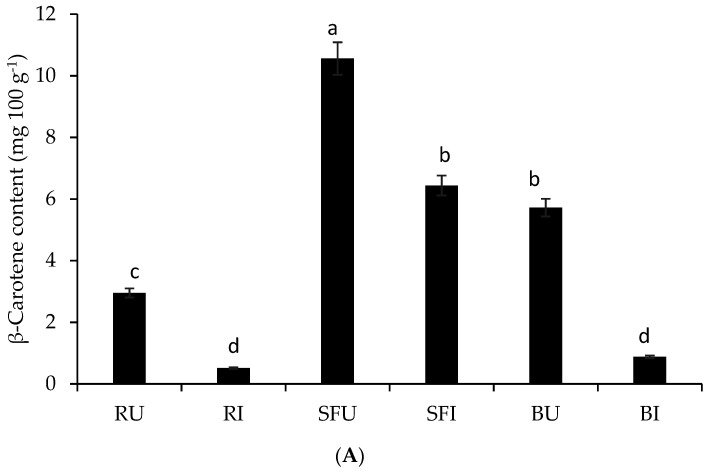

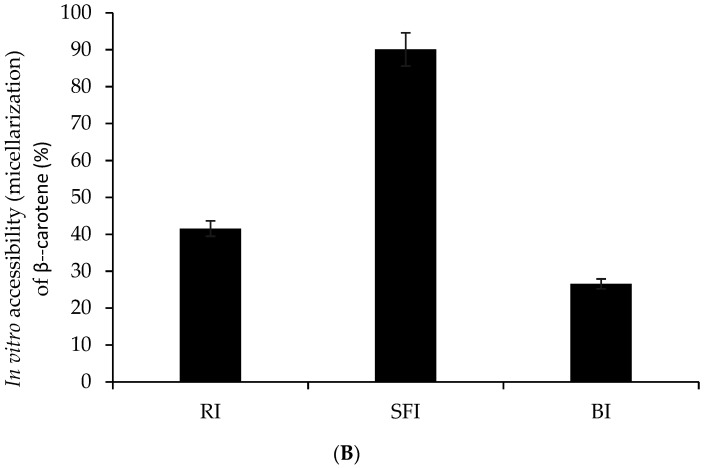
(**A**) Impact of stir-frying or boiling on the released β-carotene content from African pumpkin leaves during simulated gastrointestinal digestion compared to the whole raw leaves. RU—undigested raw leaf; RI—Intestinal digesta of raw leaves; SFU—undigested stir-fried leaves; RI—Intestinal digesta of stir-fried leaves; BU—undigested boiled leaves; BI—intestinal digesta of boiled leaves. Each bar represents the mean value of three samples per digestive stage or raw leaves; the standard deviations are included in the bars. Different alphabet letters in the same bar for African pumpkin leaves indicate significant differences at (*p* < 0.05). (**B**) In vitro bioaccessibility of β-carotene from stir-fried or boiled African pumpkin leaves compared to the whole raw leaves. RI—Intestinal digesta of raw leaves; SFI—Intestinal digesta of stir-fried leaves; BI—Intestinal digesta of boiled leaves. Each bar represents the mean value of three samples per digestive stage or raw leaves. Standard deviations are included in the bars. Different alphabet letters in the same bar for African pumpkin.

In comparison to boiled (5.72 mg 100 g^−1^) or raw (2.95 mg 100 g^−1^) leaves, β-carotene bioaccessibility was significantly increased in stir-fried leaves (10.56 mg 100 g^−1^) (Figure 3A). The chromatograms illustrating the changes in the *β*-carotene related stir-fry and boiled leaves compared to the raw leaves are shown in Supplementary Figure S2. We previously observed an increase in total carotenoid content after thermal cooking treatments [7]. The process of absorption begins with the release of carotenoids from food [39]. Several factors affect the release and incorporation of carotenoids into micelles, including household cooking, the addition of oils during cooking, the specific matrix and the type of carotenoid involved [40]. Moreover, there was a significant increase in the released and accessible β-carotene quantities from stir-fried leaves compared to raw or boiled leaves (Supplementary Figure S2). The stir-fried leaves showed 90.07% in vitro bioaccessibility of β-carotene, while boiled leaves showed 41.55%, and the raw leaves had the least at 26.58% (Figure 3B). The mechanical disruption of the green leafy vegetable matrices during stir-frying or boiling may have influenced the release of β-carotene compared to raw leaves, resulting in significantly higher values in both stir-fried and boiled undigested samples; Eriksen et al. [40] made a similar observation. Using olive oil (fat) during stir-frying could have improved the retention of β-carotene in the leaf surface. In addition, Eriksen et al. [40] found that fat combined with steaming led to a 35% increase in the liberation of β-carotene in spinach. The β-carotene availability in terms of the Recommended Daily Allowance (RDA), for men above 14 years, released in the micellar by boiling, stir-frying or in the raw African pumpkin leaves was 4.72%, 59.6% and 8.15%, respectively. For women above 14 years, raw, boiled and stir-fried African pumpkin leaves’ micellar fraction contributed 6.1%, 10.5% and 76.7% of the RDA, respectively, per 100 g leaves.

## 4. Conclusions

Understanding how household cooking methods affect the bioaccessibility of bioactive metabolites and β-carotene from green leafy vegetables can greatly enhance consumers’ health benefits. Our results confirmed that the bioaccessibility of bioactive metabolites and β-carotene from African pumpkin leaves depends on the type of household cooking method. In addition, this study recommends stir-frying African Pumpkin leaves, based on the findings, for the release and bioaccessibility of most bioactive phenolic metabolites and β-carotene rather than boiled or raw leaves. Furthermore, stir-frying African Pumpkin leaves enhanced the antioxidant activity of dialysable digesta and inhibited the carbohydrate hydrolysing enzymes. Hence, this study aims to provide consumers and chefs in the African culinary sector with a suitable household method for cooking African vegetables. However, co-digestion of African pumpkin leaves with other foods that it is normally consumed with may depict the true picture of the bioaccessibility of phytonutrients. Results obtained from this study are also relevant for dietitians and for future nutritional recommendations.

## Figures and Tables

**Table 1:** The influence of stir-frying and boiling household cooking methods on bioactive metabolites and antioxidant activity after in vitro gastrointestinal digestion of African pumpkin leaves (*Momordica balsamina* L.) compared to whole raw leaves.

	Raw Whole Leaves				Stir-Fried Leaves				Boiled Leaves			
Bioactive Metabolites (mg 100 ^g −1^)	RU	RG	RI	RD	SFU	SFG	SFI	SFD	BU	BG	BI	BD
Methylquinic acid	0.72 ± 0.13 *^,i^	578.24 ± 0.6 ^a^	554.45 ± 0.4 ^b^	2.34 ± 0.57 ^g^	0.06 ± 0.01 ^k^	265.43 ± 0.97 ^c^	223.25 ± 0.7 ^d^	0.74 ± 0.13 ^i^	0.26 ± 0.05 ^j^	62.56 ± 0.97 ^f^	74.82 ± 3.3 ^e^	1.20 ± 0.1 ^h^
Pseudolaroside A	44.90 ± 0.2 ^f^	210.70 ± 0.9 ^d^	797.65 ± 0.7 ^a^	127.15 ± 0.87 ^e^	123.43 ± 0.4 ^e^	453.06 ± 0.42 ^c^	592.41 ± 0.1 ^b^	34.79 ± 0.43 ^g^	28.20 ± 0.04 ^h^	20.38 ± 0.64 ^i^	23.17 ± 1.4 ^i^	4.59 ± 0.6 ^j^
β-D-glucosyl-2-coumarate (Melilotoside)	2.75 ± 0.4 ^j^	11.48 ± 0.3 ^g^	13.94 ± 0.8 ^g^	15.54 ± 0.8 ^e^	5.77 ± 1.03 ^i^	29.38 ± 0.94 ^b^	35.11 ± 0.34 ^a^	22.40 ± 0.36 ^c^	1.11 ± 0.20 ^k^	7.07 ± 2.21 ^h^	17.21 ± 0.4 ^d^	0.40 ± 0.1 ^l^
4 caffeoylquinic acid (Cryptochlorogenic acid)	1.20 ± 0.21 ^k^	68.48 ± 0.23 ^e^	92.68 ± 0.35 ^d^	2.94 ± 0.20 ^j^	92.19 ± 0.4 ^d^	835.73 ± 0.26 ^b^	925.81 ± 0.41 ^a^	20.05 ± 1.40 ^g^	13.82 ± 0.4 ^h^	40.03 ± 0.83 ^f^	108.32 ± 0.6 ^c^	9.00 ± 1.35 ^i^
Cis 4-coumaroylquinic acid	6.01 ± 1.0 ^h^	113.05 ± 0.1 ^c^	105.45 ± 0.8 ^d^	2.89 ± 0.20 ^j^	18.15 ± 0.2 ^g^	286.20 ± 0.73 ^b^	338.27 ± 0.06 ^a^	7.11 ± 0.50 ^h^	2.21 ± 0.40 ^j^	32.60 ± 0.28 ^f^	53.25 ± 0.15 ^e^	3.34 ± 0.5 ^i^
Trans-4-coumaroylquinic acid	3.18 ± 0.5 ^h^	14.90 ± 04 ^f^	73.47 ± 0.28 ^c^	2.98 ± 0.21 ^i^	21.20 ± 0.7 ^d^	112.37 ± 0.09 ^b^	275.00 ± 0.11 ^a^	4.16 ± 0.74 ^g^	2.47 ± 0.44 ^i^	15.00 ± 1.41 ^f^	17.26 ± 0.18 ^e^	0.80 ± 0.1 ^j^
Cis-4-feruloylquinic acid	273.6 ± 0.8 ^f^	2351.59 ± 0.6 ^b^	2593.32 ± 0.5 ^a^	162.92 ± 0.37 ^h^	178.7 ± 0.9 ^g^	2044.65 ± 0.2 ^d^	2164.70 ± 0.72 ^c^	75.05 ± 0.24 ^i^	47.49 ± 0.48 ^j^	250.15 ± 0.34 ^f^	375.42 ± 0.63 ^e^	19.38 ± 0.9 ^k^
Quercetin-3-rutinoside (Rutin)	31.86 ± 0.9 ^g^	230.82 ± 0.31 ^d^	277.95 ± 0.06 ^c^	5.07 ± 0.22 ^j^	73.66 ± 0.15 ^e^	665.89 ± 0.08 ^b^	746.56 ± 0.48 ^a^	38.68 ± 0.70 ^g^	38.12 ± 0.8 ^g^	50.95 ± 0.93 ^f^	21.71 ± 0.84 ^i^	1.86 ± 0.2 ^k^
Trans-4-feruloylquinic acid	44.52 ± 095 ^f^	156.33 ± 0.31 ^d^	308.26 ± 08.78 ^b^	61.59 ± 0.30 ^e^	47.27 ± 0.44 ^f^	287.93 ± 0.63 ^c^	341.59 ± 0.64 ^a^	17.20 ± 0.20 ^h^	3.55 ± 0.63 ^k^	13.59 ± 0.75 ^i^	27.23 ± 0.8 ^g^	6.41 ± 0.9 ^j^
Quercetin 3-galactoside	4.05 ± 0.72 ^f^	41.35 ± 0.96 ^c^	84.21 ± 0.44 ^b^	3.10 ± 0.75 ^g^	37.47 ± 0.6 ^d^	322.22 ± 0.97 ^a^	337.18 ± 0.09 ^a^	8.18 ± 0.57 ^e^	2.83 ± 0.51 ^h^	3.48 ± 0.72 ^g^	8.61 ± 0.74 ^e^	1.57 ± 0.23 ^i^
Kaempferol-O-rutinoside (Nicotiflorin)	42.05 ± 0.51 ^f^	455.43 ± 0.42 ^d^	715.34 ± 0.55 ^a^	13.83 ± 0.97 ^g^	77.42 ± 0.82 ^e^	629.04 ± 0.15 ^b^	513.64 ± 0.76 ^c^	12.76 ± 0.89 ^h^	9.23 ± 1.65 ^i^	6.07 ± 1.37 ^j^	9.44 ± 0.54 ^i^	1.57 ± 0.23 ^k^
Isorhamnetin 3-O-robinoside (Keioside)	7.59 ± 1.36 ^g^	112.35 ± 0.01 ^d^	155.23 ± 0.43 ^c^	1.59 ± 0.38 ^i^	45.32 ± 0.09 ^e^	330.99 ± 01.00 ^b^	352.25 ± 87.35 ^a^	9.89 ± 0.77 ^f^	2.27 ± 0.41 ^h^	1.91 ± 0.87 ^i^	1.53 ± 0.58 ^i^	0.24 ± 0.04 ^j^
Rhamnetin-3-O-glucoside	1.05 ± 0.19 ^f^	12.61 ± 0.08 ^d^	14.54 ± 0.24 ^c^	0.75 ± 0.18 ^h^	10.08 ± 1.80 ^e^	64.42 ± 0.91 ^b^	74.36 ± 38.68 ^a^	1.27 ± 0.23 ^f^	0.47 ± 0.08 ^i^	0.86 ± 0.35 ^g^	0.38 ± 0.37 ^j^	nd
Phenethyl rutinoside	17.41 ± 3.11 ^c^	2.49 ± 0.29 ^g^	14.38 ± 0.07 ^d^	3.60 ± 0.25 ^f^	11.07 ± 0.98 ^e^	22.83 ± 0.50 ^b^	35.23 ± 4.75 ^a^	1.64± 0.29 ^i^	1.82 ± 0.33 ^h^	3.05 ± 2.07 ^f^	2.84 ± 0.89 ^g^	1.00 ± 0.15 ^j^
**Antioxidant activity**												
FRAP mmol TEAC g ^−1^	3.65 ± 0.01 ^h^	6.41 ± 0.01 ^f^	11.69 ± 0.02 ^c^	1.20 ± 0.02 ^j^	9.73 ± 0.02 ^d^	12.92 ± 0.01 ^b^	16.40 ± 3.10 ^a^	2.60 ± 0.05 ^i^	1.38 ± 0.01 ^j^	5.57 ± 0.07 ^g^	7.75 ± 0.01 ^e^	1.18 ± 0.06 ^j^
DPPH IC_50_ (mg mL^−1^)	0.53 ± 0.01 ^b^	0.30 ± 0.00 ^d^	0.18 ± 0.05 ^f^	0.68 ± 0.02 ^a^	0.22 ± 0.01 ^e^	0.16 ± 0.02 ^f^	0.13 ± 0.00 ^g^	0.54 ± 0.01 ^b^	0.40 ± 0.01 ^c^	0.32 ± 0.00 ^d^	0.28 ± 0.00 ^e^	0.64 ± 0.05 ^a^
ABTS IC_50_ (mg mL^−^^1^)	0.58 ± 0.01 ^d^	0.31 ± 0.03 ^f^	0.15 ± 0.01 ^h^	0.80 ± 0.01 ^a^	0.21 ± 0.02 ^g^	0.14 ± 0.00 ^h^	0.08 ± 0.06 ^i^	0.64 ± 0.02 ^c^	0.48 ± 0.02 ^e^	0.34 ± 0.00 ^f^	0.28 ± 0.01 ^g^	0.71 ± 0.01 ^b^

Values are mean ± standard deviation *, and means followed by a different letter within the row are significantly different (*p* < 0.05); RU—Raw whole leaf undigested; BU—undigested boiled leaf; SFU—undigested stir-fried leaf; RG—gastric digesta of raw leaf; BG—gastric digesta of boiled leaf; SFG—gastric digesta of stir-fried leaf; RI—Intestinal digesta of raw leaf; BI-Intestinal digesta of boiled leaf; SFI—Intestinal digesta of stir-fried leaf; RD—dialysable digesta of raw leaves; BD—dialysable digesta of boiled leaves; SFD—dialysable digesta of stir-fried leaves.

**Table 2:** Effects of household cooking methods and simulated gastrointestinal digestion on the inhibitory effect of African pumpkin leaves (*Momordica balsamina* L.) on carbohydrate hydrolysing enzymes.

Treatment	α-Amylase IC_50_ mg mL^−1^	α-Glucosidase IC_50_ mg mL^−1^
Raw whole leaves	0.24 ± 0.04 ^g^	0.41 ± 0.04 ^e^
Raw leaves at gastric phase	0.36 ± 0.50 ^e^	0.43 ± 0.10 ^e^
Raw leaves at intestinal phase	0.28 ± 0.40 ^f^	0.15 ± 0.20 ^h^
Raw leaves at dialysis phase	1.15 ± 0.15 ^b^	0.89 ± 0.04 ^b^
Stir-fried leaves	0.14 ± 0.03 ^h^	0.25 ± 0.02 ^g^
Stir-fried leaves at gastric phase	0.13 ± 0.20 ^h^	0.31 ± 0.00 ^f^
Stir-fried leaves at intestinal phase	0.08 ± 0.30 ^i^	0.06 ± 0.20 ^i^
Stir-fried leaves at dialysis phase	0.43 ± 0.04 ^d^	0.32 ± 0.10 ^f^
Boiled leaves	0.69 ± 0.02 ^c^	0.75 ± 0.30 ^c^
Boiled leaves at gastric phase	0.32 ± 0.10 ^e^	0.29 ± 0.20 ^g^
Boiled leaves at intestinal phase	0.27 ± 0.20 ^g^	0.16 ± 0.20 ^h^
Boiled leaves at dialysis phase	0.63 ± 0.05 ^c^	0.50 ± 0.05 ^d^
Acarbose	3.14 ± 0.13 ^a^	6.87 ±0.22 ^a^

Different alphabet letters in the same column for African pumpkin leaves indicate significant differences at (*p* < 0.05).

## Data Availability

All data are provided in the manuscript.

## Supplementary Materials

The following are available online at https://www.mdpi.com/article/10.3390/foods10112890/s1, Table S1 UPLC-QTOF/MS analyses of major metabolites detected in raw and cooked African leaves; Supplementary Figure S1 illustrating the increase in bioactive metabolites in the gastric, intestinal and dialysable digesta of stir-fried African pumpkin leaves compared to the boiled and raw leaves; Supplementary Figure S2 showing stir-fried African pumpkin (*Momordica balsamina* L.) leaves (pink) with the highest level of β-carotene (peak A), boiled African pumpkin leaves (blue) with a lower level of β-carotene, and raw leaves (black) with the lowest amount of β-carotene.
