# Supplementary material for: Cooking African Pumpkin Leaves (Momordica balsamina L.) by Stir-Frying Improved Bioactivity and Bioaccessibility of Metabolites—Metabolomic and Chemometric Approaches
Source: Foods. 2021 Nov 22;10(11):2890. doi: 10.3390/foods10112890 (PMC8621757; doi:10.3390/foods10112890)
Supplement: Supplementary file 1 [file foods-10-02890-s001.zip › foods-1449898-supplementary.pdf]

foods

MDPI

Supplementary Material

Cooking African Pumpkin Leaves (*Momordica balsamina* L.) by Stir-Frying Improved Bioactivity and Bioaccessibility of Metabolites—Metabolomic and Chemometric Approaches

Petunia Mashiane [1,2], Vimbainashe E Manhivi [2], Tinotenda Shoko [2], Retha M Slabbert [1], Yasmina Sultanbawa [3] and Dharini Sivakumar [2,3,*]

[1] Department of Horticulture, Tshwane University of Technology, Pretoria P.O. 0001, South Africa; MashianeP@tut.ac.za (P.M.); SlabbertMM@tut.ac.za (R.M.S.)

[2] Phytochemical Food Network Research Group, Department of Crop Sciences, Tshwane University of Technology, Pretoria 0001, South Africa; ManhiviVE@tut.ac.za (V.E.M.); ShokoT@tut.ac.za (T.S.)

[3] Agricultural Research Council Industrial Transformation Training Centre for Uniquely Australian Foods, Queensland Alliance for Agriculture and Food Innovation, The University of Queensland, Brisbane, QLD, Australia; y.sultanbawa@uq.edu.au

* Correspondence: SivakumarD@tut.ac.za

Citation: Mashiane, P.; Shoko, T.; Slabbert, R.M.; Sultanbawa, Y. Cooking African Pumpkin Leaves (*Momordica balsamina* L.) by Stir-Frying Improved Bioactivity and Bioaccessibility of Metabolites—Metabolomic and Chemometric Approaches. *Foods* **2021**, *10*, 2890. https://doi.org/10.3390/foods10112890

Academic Editor: Annalisa Tassoni

Received: 20 October 2021
Accepted: 17 November 2021
Published: 22 November 2021

Publisher's Note: MDPI stays neutral with regard to jurisdictional claims in published maps and institutional affiliations.

Copyright: © 2021 by the authors. Licensee MDPI, Basel, Switzerland. This article is an open access article distributed under the terms and conditions of the Creative Commons Attribution (CC BY) license (http://creativecommons.org/licenses/by/4.0/).

Table S1. UPLC-QTOF/MS analyses of major metabolites detected in raw and cooked African leaves .

Compound	RT(min)	[M-H]$^-$	Formula	Error (ppm)	MSE fragments	UV	Tentative Identification
1	7.09	205.03609	$C_7H_{10}O_7$	-3.47	173.011	230	Methylquinic acid
2	11,38	299.0764	$C_{13}H_{16}O_8$	2.82	137.024<br>93.034	283	Pseudolaroside A
3	12,44	325,0923	$C_{15}H_{18}O_8$	1.82	163.040	269	β- D- glucosyl- 2-coumarate (Melilotoside)
4	14.00	353,05099	$C_{15}H_{14}O_{10}$	1.23	191.020<br>173.009<br>135.099	326	4 caffeoylquinic acid (Cryptochlorogenic acid)
5	16.27	337.05606	$C_{15}H_{14}O_9$	1.33	191.9496<br>173.9740<br>163.0427	312	Cis 4-coumaroylquinic acid
6	16.98	337.04831	$C_{15}H_{14}O_8$	-31.09	191.019<br>173.010<br>163.145	309	Trans-4-coumaroylquinic acid
7	17.45	367.06699	$C_{16}H_{16}O_{10}$	0.22	173.009	326	4 Feruloylquinic acid
8	17.61	609.14404	$C_{27}H_{30}O_{16}$	3.40	300.028<br>271.023<br>178.982<br>151.003	351	Quercetin-3-rutinoside (Rutin)
9	17.90	367.06723	$C_{16}H_{16}O_{10}$	-0.42	173.009	326	Feruloylisocitric acid isomer
10	18.19	463.08975	$C_{21}H_{20}O_{12}$	-3.34	300.029<br>271.026<br>179.009<br>151.005	265<br>346	Quercetin 3-galactoside
11	19.23	593.15198	$C_{27}H_{30}O_{15}$	-1.32	285.040<br>163.004	253<br>352	Kaempferol-O-rutinoside (Nicotiflorin)
12	19.67	623.16125	$C_{28}H_{32}O_{16}$	0.82	315.050<br>300.026	230	Isorhamnetin 3-O-robinoside (Keioside)
13	20,39	477.09436	$C_{22}H_{22}O_{12}$	19.89	314.041<br>300.039<br>285.053	230	Rhamnetin-3-O-glucoside
14	20,68	429.17532	$C_{20}H_{30}O_{10}$	3.04	325.1318<br>161.1176	276	Phenethyl rutinoside

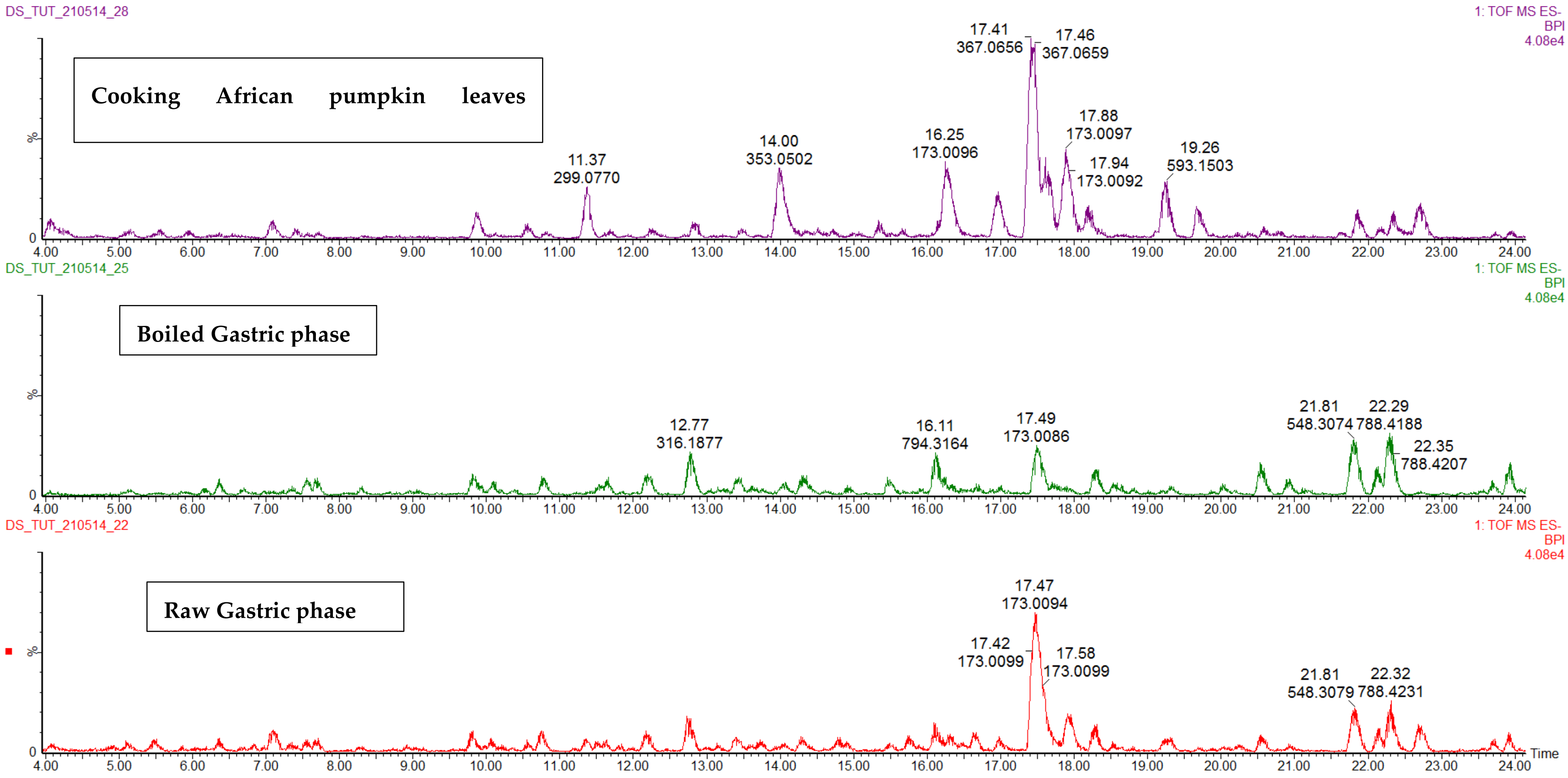
DS_TUT_210514_28
1: TOF MS ES-
BPI
4.08e4
Cooking African pumpkin leaves
17.41
367.0656
17.46
367.0659
17.88
173.0097
17.94
173.0092
16.25
173.0096
14.00
353.0502
11.37
299.0770
19.26
593.1503
DS_TUT_210514_25
1: TOF MS ES-
BPI
4.08e4
Boiled Gastric phase
12.77
316.1877
16.11
794.3164
17.49
173.0086
21.81
548.3074
22.29
788.4188
22.35
788.4207
DS_TUT_210514_22
1: TOF MS ES-
BPI
4.08e4
Raw Gastric phase
17.47
173.0094
17.42
173.0099
17.58
173.0099
21.81
548.3079
22.32
788.4231
%
0
Time
4.00
5.00
6.00
7.00
8.00
9.00
10.00
11.00
12.00
13.00
14.00
15.00
16.00
17.00
18.00
19.00
20.00
21.00
22.00
23.00
24.00

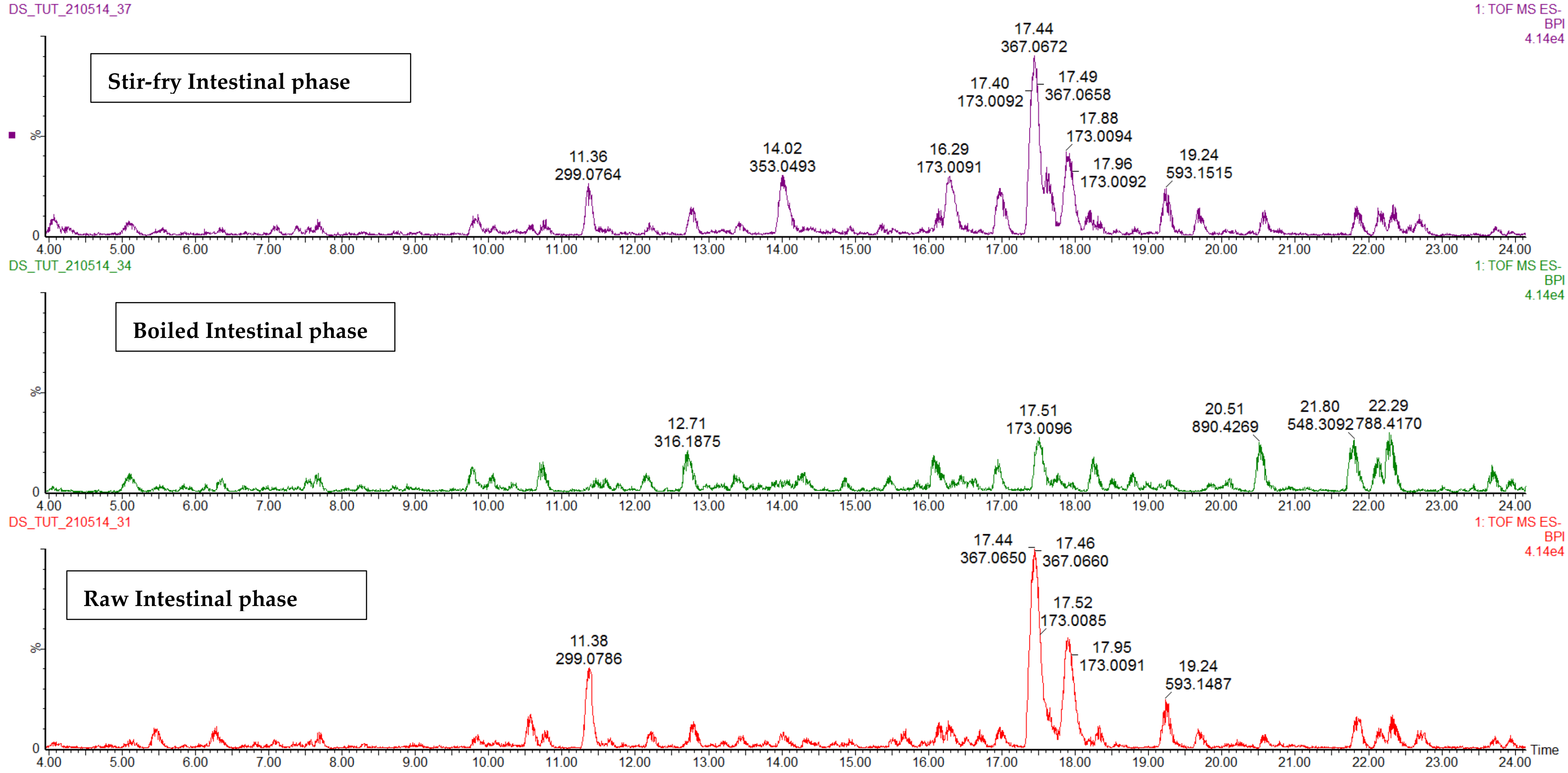
DS_TUT_210514_37
1: TOF MS ES-
BPI
4.14e4
Stir-fry Intestinal phase
11.36
299.0764
14.02
353.0493
16.29
173.0091
17.40
173.0092
17.44
367.0672
17.49
367.0658
17.88
173.0094
17.96
173.0092
19.24
593.1515
DS_TUT_210514_34
1: TOF MS ES-
BPI
4.14e4
Boiled Intestinal phase
12.71
316.1875
17.51
173.0096
20.51
890.4269
21.80
548.3092
22.29
788.4170
DS_TUT_210514_31
1: TOF MS ES-
BPI
4.14e4
Raw Intestinal phase
11.38
299.0786
17.44
367.0650
17.46
367.0660
17.52
173.0085
17.95
173.0091
19.24
593.1487
%
0
Time
4.00 5.00 6.00 7.00 8.00 9.00 10.00 11.00 12.00 13.00 14.00 15.00 16.00 17.00 18.00 19.00 20.00 21.00 22.00 23.00 24.00

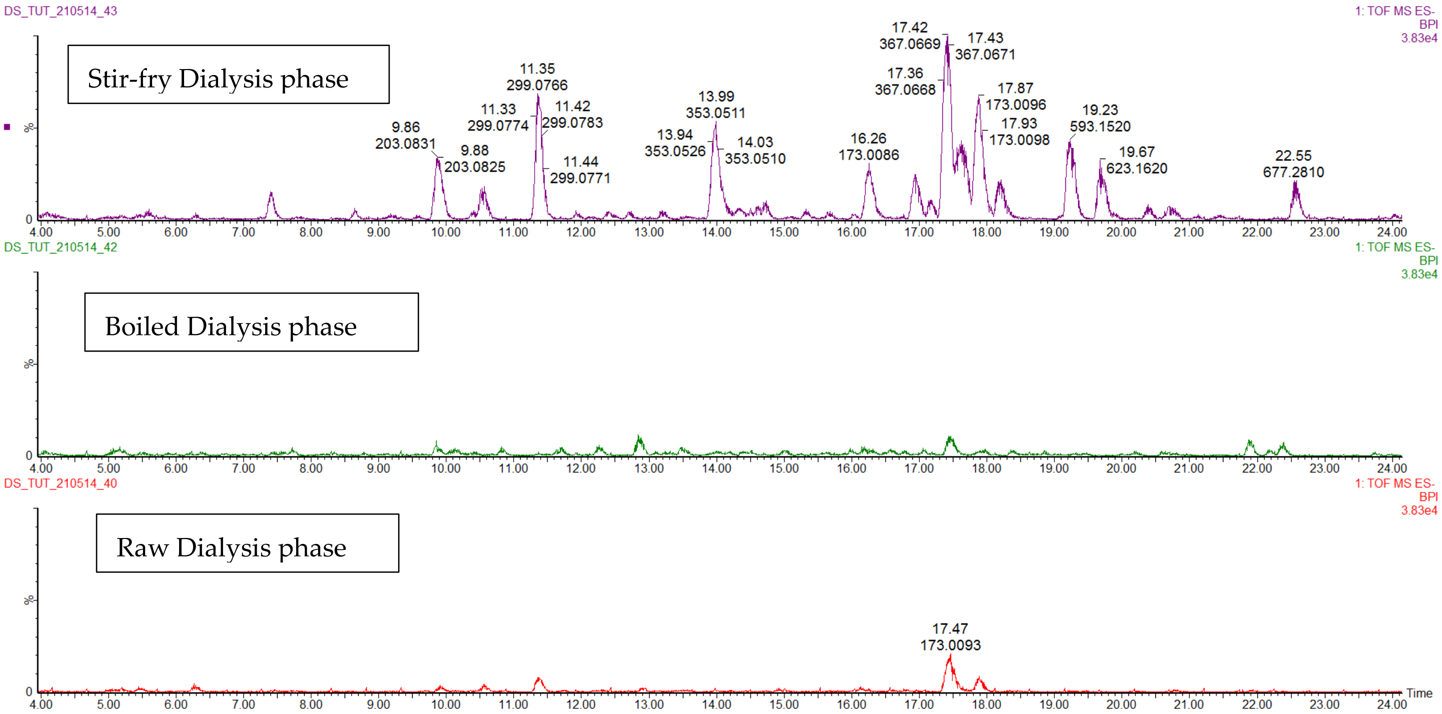

Figure S1. illustrating the increase of bioactive metabolites in the gastric, intestinal and dialysable digesta of stir-fried African pumpkin leaves compared to the boiled and raw leaves. .

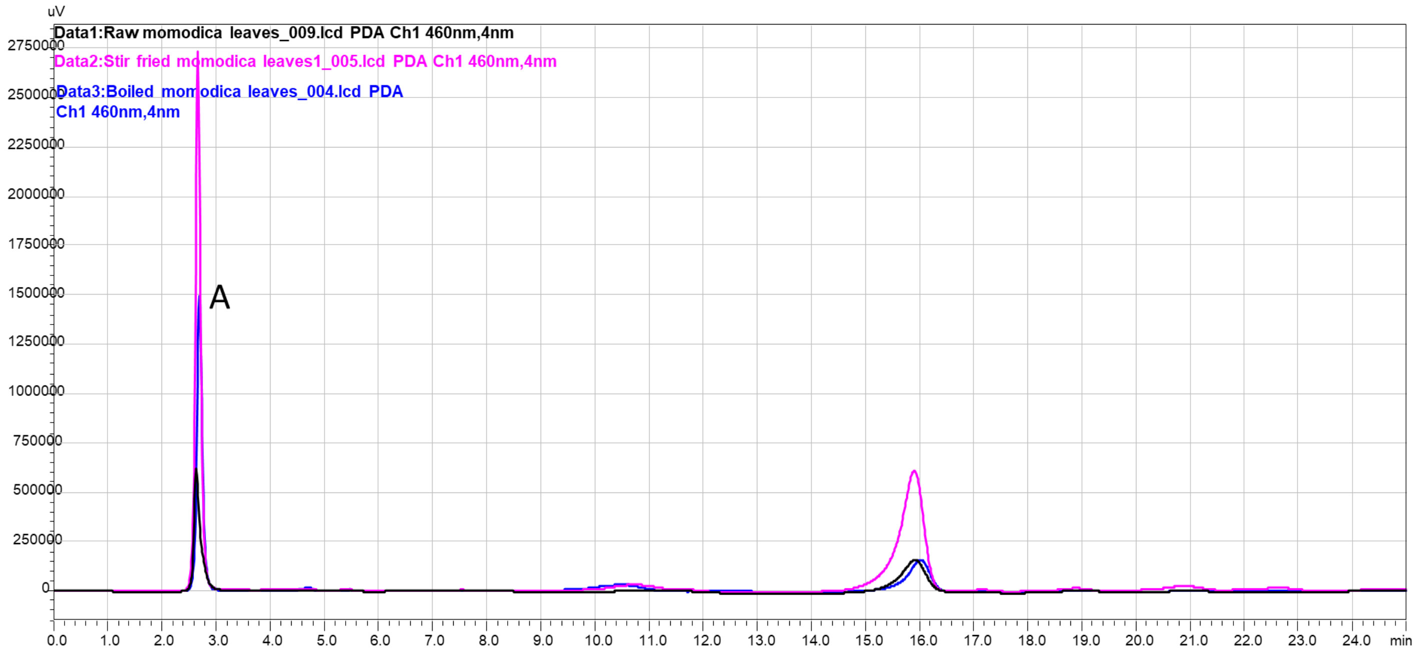

Figure S2. Showing stir fried African pumpkin (Momordica balsamina L.) leaves (pink) with the highest level of β-carotene (peak A), boiled African pumpkin leaves (blue) with a lower level of β-carotene, and raw leaves (black) with the lowest amount of β-carotene.
